# Laboratory Diagnosis of a NZ7-like Orf Virus Infection and Pathogen Genetic Characterization, Particularly in the *VEGF* Gene

**DOI:** 10.3389/fvets.2020.00538

**Published:** 2020-09-17

**Authors:** Yongzhong Yu, Xuyang Duan, Yuanyuan Liu, Jinzhu Ma, Baifen Song, Zhengxing Lian, Yudong Cui

**Affiliations:** ^1^College of Biological Science and Technology, Heilongjiang Bayi Agricultural University, Daqing, China; ^2^College of Animal Science and Technology, China Agricultural University, Beijing, China

**Keywords:** orf virus, isolation, identification, genetic characterization, VEGF genotype

## Abstract

Orf is a widespread contagious epithelial viral disease found particularly in most sheep breeding countries in the world. Recently, an orf virus (ORFV) strain OV-HLJ05 was isolated from an outbreak in northeast China. Three genes of interest including ORFV011 (B2L), ORFV059 (F1L), and ORFV132 (VEGF) of ORFV, were recruited to identify and genetically characterize this newly isolated virus. Amino acid (aa) sequence compared with the ORFV references listed in GenBank, both B2L and F1L of OV-HLJ05 showed less microheterogeneity from their references. In contrast, the VEGF gene was included in the NZ7-VEGF like group as previously considered by Mercer in 2002. Unexpectedly, further multiple VEGF matches were made, using 34 published sequences from China and India, resulting in 27 strains of the NZ7 members. Based on Karki's report in 2020, NZ7-VEGF like viruses are emerging more and more frequently in these two countries, damaging the Asian sheep industry. Obvious heterogeneity with the NZ2, insertion of two oligopeptides TATI(L)QVVVAI(L) and SSSS(S) motif were found in the NZ7-like VEGF protein. These VEGFs are divided mainly into two types and a significant increase in the number of hydrogen bonds within the NZ7-like VEGF dimers was observed. The NZ7-like ORFV apparently favors the goat as a host and an emphasis on this in future epidemiological and pathological studies should be considered, focusing on the NZ7-like virus.

## Introduction

Orf is an animal pustular dermatitis and an epitheliotrophic contagious disease directly caused by the orf virus (ORFV) with a worldwide distribution (1, 2). This viral skin disease commonly affects sheep, goats, and some other ruminants and has a zoonotic potential in humans who are exposed to a contaminated workplace (3–6). Clinically, orf disease progresses from erythema to macule, papule, and vesicle formation and then from pustules to thick scabs. Severely affected animals may lose weight and become more susceptible to secondary bacterial infections (7). Prolonged infection and increased severity are associated with often severe secondary bacterial infection. More usually, minor staphylococcal infection is a frequent occurrence, but mortality rates can be over 5% in infected herds (8). Higher mortality occurs frequently in lambs or kids during the lactation period due to dehydration and starvation, as the pain and distortion of the lips and mouth reduces sucking (8, 9). Because orf has serious economic and environmental impacts in most sheep-feeding countries in the world, it is important to characterize the pathogen of any outbreak in breeding livestock. It is also especially important to determine regional ORFV strains, to predict the risk of outbreak in affected developing countries such as India and China, to improve prevention and control management.

ORFV is a prototype member of genus *Parapoxvirus* (PPV) with a G+C content about 64 percent in the genome (10). The virus has a linear double-stranded closed DNA of nearly 150 kbp in genome length containing 130 to 132 putative genes, with 88 genes conserved in PPVs (1, 10). These genes are responsible for viral replication and the composition of the fixed asset in the center of the genome, while two highly variable regions are located in the closed terminal ends of the viral genome, which encode proteins required for viral invasion (11) or immune evasion (12).

At present there are abundant ORFV isolate sequences published in GenBank, with some full length genome data available, with six of them extensively researched previously such as ORFV-NZ2 (10, 13), ORFV-NZ7 (14, 15), ORFV- SA00, and ORFV-IA82 (16), ORFV-D_1701_ (11), a human biopsy-derived virus ORFV-B029 (partial genome) (17) and eight new ORFV strains from China including ORFV-NA1/11 (18); ORFV-GO, -NP, -YX, and -SJ1 (19); NA17 (*Accession number*:*MG674916*) (20), Shanxi (*Accession number*:*AEN14425*) and Fujian-XP (*Accession number*:*AIZ05258*). These strains may provide many references for evaluating an emerging pathogen from any orf epidemic.

For more accurate diagnosis of orf in the lab, both conventional PCR and real-time PCR methods are used for higher specificity and sensitivity in the detection of viral ORFV pathogens. These techniques have been developed based on the major membrane glycoprotein gene *B2L* (ORFV011) (6, 21, 22) or on the DNA polymerase gene (23). Generally, *B2L* with conserved quality in different PPV species is used as a common and precise marker for examining a virus with its genetic stability, such as ORFV, bovine popular stomatitis virus (BPSV), pseudocowpox virus (PCPV) and parapoxvirus of red deer in New Zealand (PVNZ) (21). Parapox virus can therefore be confirmed by the *B2L* gene on a molecular level in the laboratory, because GenBank can provide abundant *B2L* reference information for researchers (24). Though the *B2L* gene is adopted for the genetic phylogenetic investigation of ORFV (25–28), *B2L* gene data alone is not sufficient to confirm a viral species.

The secondary gene of interest for pathogen investigation is the *F1L* gene (ORFV059) that encodes an envelope protein to exploit a subtle interaction between virus and host, then initiates viral invasion by binding to heparan-sulfate sensors outside the host cells (29). *The* F1L protein, as the main immunogenic protein of ORFV, is transcribed in the mid-late stage of the viral infection period and can bind to glycosaminoglycan (GAG) on the mammalian cell (30). Several functional regional and amino acid motifs are also found in F1L proteins, including a proline-rich region (PRR) and KGD motif, unique motifs in ORFV, and some conservative motifs such as GAG, D/ExD, and Cx3C among the *Poxviridae* family, which are apparent in sequence alignment of ORFVs (31).

The ORFV132 gene has been of interest because it encodes a vascular endothelial growth factor (VEGF) of ORFV which has a direct responsibility for the extensive vascular hyperplastic lesions (32). As a result, the ORFV132 gene is expressed early during infection of ORFV (15); but it has not been found in other poxviruses. The *VEGF* gene therefore plays a unique role in virulence analysis, although it is not the only virulence factor that has been identified. The *VEGF* genes among PPVs show numerous variants which can share only 41 to 61% amino acid sequence identity (16). For ORFVs, two genotype groups were classed by the NZ2- and NZ7-VEGF like genes which show little DNA homology to each other, whereas the flanking sequences are over 98% homologous (15). The reported sequence variations might reflect the genetic drift of the *VEGF* gene although the rate of drift seems greater than generally seen in poxvirus genes (33). More recently, Karki et al., reported that the majority of Indian ORFV isolates showed 78.4 to 99.3% amino acid identity with each other in the VEGF gene, even like the NZ7-like VEGF (34). Given that different ORFV isolates from the world show these two genotypes in VEGF, this study places the emphasis on the regional distribution of VEGF genotype, to explain its genetic characteristics and clinical features related to the environment and species.

This paper reports on a new ORFV isolate from the northeast of China. Genetic studies of three genes mentioned above and the VEGF molecular structure observation with high resolution have been performed following the virological identification.

## Materials and Methods

### Clinical Case and Virus Isolation

During an outbreak in the autumn of 2017, a local farmer reported that five young Boer goats were found to be affected by a contagious skin disease with obvious lesions in the oral cavity or lips, but lesion material had only been collected from a 6-month-old kid, that died in an isolation area.

In this flock of over 200 Boer goats, there were no other domestic mammals. The sheep pen was simple, with only guardrails and a roof and sanitary conditions were poor. It was speculated that the outbreak may have been related to stock bought in from other provinces in China several months ago. According to the farmer, none of the animals in this flock had been given orf vaccine before this outbreak but they had been treated with externally applied agents such as gentian violet. The majority of affected animals had recovered spontaneously except the single death.

For virus isolation, Madin-Darby bovine kidney (MDBK) and human keratinocyte cell line (HaCaT) cells (both of these cells are cryopreserved in liquid nitrogen in our laboratory) were cultured separately in DMEM containing 10% fetal bovine serum (FBS), 100 U/mL penicillin and 100 μg/mL streptomycin at 37°C with 5% CO_2_ (35). As per Yu's protocol, a confluent monolayer of MDBK cells were inoculated with some viral supernatant (36). When 70 to 80% cytopathic effect (CPE) was reached, the cells were harvested followed by freezing at −80°C. The virions were further purified by sucrose gradient ultra-centrifugation. A major virus band was obtained after centrifugation of virus infected MDBK cells in the 32–36% sucrose gradient. Electron microscopy (EM) investigation was completed by negative staining.

### EM for Ultrastructural Analyses

The viral samples were assayed immediately as described by Yu et al. (36). Lead citrate was used to make the contrast background, to distinguish ORFV virion with outline geometrical characters and some surface structures.

### Immunofluorescence Microscopy

Cells were fixed with 4% paraformaldehyde for 20 min, then incubated in PBS containing 0.2% Triton-X100 for permeabilizing. After three washes in PBS, the cells were incubated with 1% bovine serum albumin (BSA) solution for 30 min. The fixed cells were incubated with 2E4 monoclonal antibody (mAb) (anti-B2L) (hybridoma cells of 2E4 mAb are cryopreserved in liquid nitrogen in our laboratory) for 1 h at 37°C. After three washes with PBS, secondary antibodies were introduced to bind 2E4 mAb at a 1:500 dilution in PBS for 30 min. Anti-mouse Ig conjugated with fluorescein isothiocyanate (FITC) (Sigma-Aldrich) was used as the secondary antibody and images were taken using a Leica fluorescence microscope.

### DNA Clone, Sequencing, and Phylogenetic Analysis

Viral DNA was prepared based on commercial kits protocols for polymerase chain reaction (PCR) amplification. Primers used in this study involving in B2L, F1L, and VEGF genes, were designed referencing the ORFV-NZ2 strain (*Accession number: DQ184476*). In addition, the alternative primers of VEGF gene were designed according to the ORFV-NZ7 strain (*Accession number: S67522*) (Table 1). After DNA amplification and purification, the target genes were each inserted into PMD18T vectors and the recombinant plasmids of positive clone were sent to Sangon Biological Engineering Technology and Services Co. Ltd. (China), for sequencing. For genetic relationship analysis of B2L, F1L, and VEGF genes among some reference strains available on GenBank at the amino acid (aa) level, a series of aa composition comparisons of the isolates were conducted using the DNAStar program (DNAStar, Inc. USA). All different source sequences in the world were included in Table 2 and molecular phylogeny and the genetic relationship of this ORFV strain with others were calculated as referenced by Yu et al. (36).

**Table 1:** PCR primers designed referencing to the popular strains of ORFV.

**Name**	**Nucleotide**	**Endonuclease**	**Reference**
B2L-F	CG*GGATCC*ATGTGGCCGTTCTCCTCCATC	*BamH* I	OV-NZ2(*DQ184476*)
B2L-R	CCC*AAGCTT*TTAATTTATTGGCTTGCAGAACTC	*Hind* III	OV-NZ2(*DQ184476*)
F1L-F	CG*GAATCC*ATGGATCCAC CCGAAATCACG	*EcoR* I	OV-NZ2(*DQ184476*)
F1L-R	CCC*AAGCTT*TCACACGATGGCCGTGACC	*Hind* III	OV-NZ2(*DQ184476*)
VEGF-F	CGC*GGATCC*ATGAAGTTGCTCGTCGGCATA	*BamH* I	OV-NZ2(*DQ184476*)
VEGF-R	CCC*AAGCTT*CTAGCGGCGTCTTCTGGGCG	*Hind* III	OV-NZ2(*DQ184476*)
VEGF-F'	GC*GGATCC*ATGAAGTTAACAGCTACCATA	*BamH* I	OV-NZ7(*S67522*)
VEGF-R'	CCC*AAGCTT*TCGTCTAGGTTCCCTAGT	*Hind* III	OV-NZ7(*S67522*)

**Table 2:** Part of VEGF genes published in GenBank recent years were used in this study.

**No**.	**Name of strain or isolate**	**Country**	**Host**	**Collection date**	**GenBank Accession No**.	**Target gene**
1	OV-SA00▴	USA	Goat	2004	AY386264	VEGF
2	OV-NZ2▴	New Zealand	Sheep	2006	DQ184476	VEGF
3	OV-NZ7▴	New Zealand	Sheep	2016	S67522	VEGF
4	ORFV Mukteswar/09	India	Sheep	2010	GU139358	VEGF
5	Cam/09	India	Camel	2010	GU460373	VEGF
6	ORFV/Mukteswar/59/05/Goat/P51	India	Goat	2018	MF414681	VEGF
7	ORFV/Mukteswar/59/05/Goat/P6	India	Goat	2018	MF414682	VEGF
8	ORFV/Meghalaya/SP45/Goat/2003	India	Goat	2018	MF414683	VEGF
9	ORFV/Shahjahanpur/82/Goat/2004	India	Goat	2018	MF414684	VEGF
10	ORFV/Jalandhar/SP41/Goat/2007	India	Goat	2018	MF414685	VEGF
11	ORFV/Bangalore/89/05/Goat	India	Goat	2018	MF414686	VEGF
12	ORFV/Hyderabad/25/Sheep/2006	India	Sheep	2018	MF414687	VEGF
13	ORFV/Gujarat/SP26/Goat/2006	India	Goat	2018	MF414688	VEGF
14	ORFV/Assam/LK/Goat/2014	India	Goat	2018	MF414689	VEGF
15	ORFV/Bhopal/Goat	India	Goat	2018	MF414690	VEGF
16	NP	China	Goat	2015	KP010355	VEGF
17	NA17	China	Goat	2015	MG674916	VEGF
18	Shanxi	China	Ovis aries	2016	AEN14425	VEGF
19	Fujian-XP	China	Goat	2016	AIZ05258	VEGF
20	NA1/11	China	Sheep	2014	JQ663432	VEGF
21	Xinjiang1	China	Goat	2013	KF666562	VEGF
22	SY17	China	Sheep	2018	MG712417	VEGF
23	OV-HN3/12	China	Sheep	2018	KY053526	VEGF
24	Shihezi2/SHZ2	China	Goat	2013	KF726849	VEGF
25	Shihezi3/SHZ3	China	Goat	2013	KF726850	VEGF
26	DG	China	Goat	2016	KM675376	VEGF
27	YX	China	Goat	2016	KM675382	VEGF
28	XD	China	Goat	2016	KM675377	VEGF
29	FQ	China	Goat	2016	KM675383	VEGF
30	GT	China	Goat	2016	KM675384	VEGF
31	SL	China	Goat	2016	KM675385	VEGF
32	DS	China	Goat	2016	KM675386	VEGF
33	GS	China	Goat	2016	KM675387	VEGF
34	SJ2	China	Goat	2016	KM675388	VEGF
35	GO	China	Goat	2016	KM675380	VEGF
36	SJ1	China	Goat	2016	KM675381	VEGF
37	OV-HLJ05⋆	China	Goat	2019	MK317956	VEGF

*Black pentastar means the orf virus isolate in this paper; Black triangle means the important reference strain*.

### Homologous Modeling Analyzes on the Present VEGFs

Prediction of the three dimensional structure of the VEGF-variants of ORFVs was modeled using SWISS-MODEL online program (*http://swissmodel.expasy.org*). The structure of the VEGF-variants, including protein subunits A and B, chain A and chain B, were viewed and aligned using the *UCSF Chimera version 1.1*. where the function of Iterative Magic Fit was used for energy minimization and the alignments were manually optimized. Ramachandran plots for the viral VEGF models were compared to determine if the viral models contained residues that did not conform to acceptable ϕ and/or ψ angles (33).

## Results

### A Case of Orf

**Figure 1:**
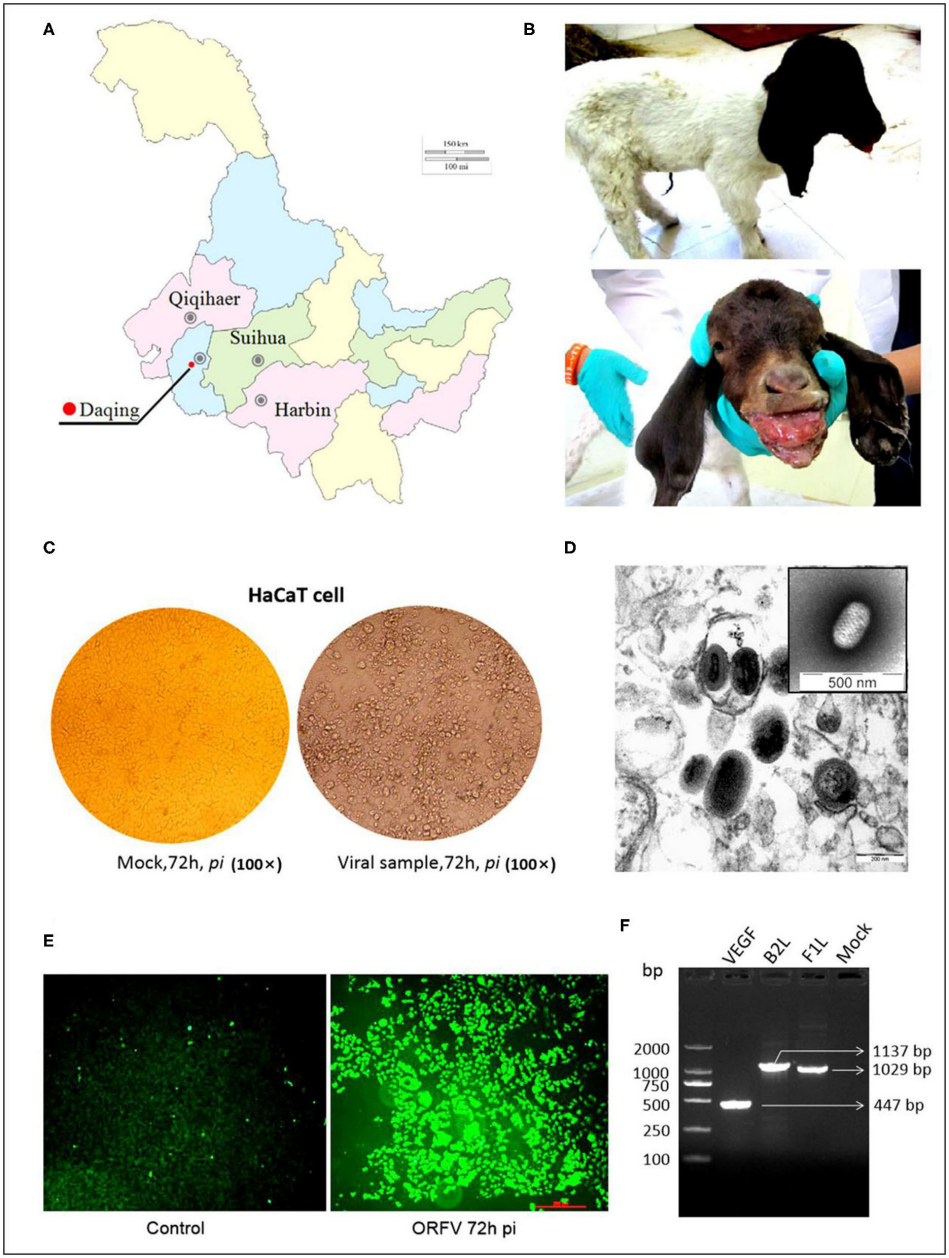
ORFV isolation from an orf outbreak in the northeast of China. **(A)** Picture indicates the geographical location of the outbreak in China, Sep 2017. **(B)** Typical clinical lesions of orf in a Boer goat kid. The severe tumid lesions in his mouth region showed more pejorative anabrosis nidus. **(C)** Cytopathic effect on HaCaT cells infected by ORFV sample. Mock-infected HaCaT cells appeared an ordered fashion after 72 h, while HaCaT cells infected with the supernatants became ragged, appearing rounded and pyknotic, with retraction of the cell membranes from surrounding cells at 72 h *pi* (×200). **(D)** The virions in MDBK cell revealed the typical ovoid shape when observed by electron microscopy. **(E)** The result of the immunofluorescence assay. Anti-B2L monoclonal antibody (mAb) (2E4, 1:200) was used as the primary antibody. **(F)** Amplification of *B2L* gene, *F1L* gene, and *VEGF* gene. Lane 1:DL2000 DNA Marker (bp); Lane 2: *VEGF* gene (447 bp); Lane 3: *B2L* gene (1137 bp); Lane 4: *F1L* gene (1029 bp); Lane 5: Mock.

For descriptive epidemiology of this outbreak in Daqing city of Heilongjiang province of China (Figure 1A), this case was briefly reported in the materials and methods. Affected animals had visible scars from clinical lesions of contagious ecthyma in their lips and angulus oris. The subject kid had developed severe anabrosic lesions in its mouth region, prior to death (Figure 1B). Clinical material such as scabs were gathered from the dead Boer kid for laboratory virus isolation.

### ORFV Isolation and Identification

In the laboratory, a sterile suspension was prepared using the clinical material to inoculate MDBK and HaCaT cell monolayers, until the CPE was observed on day 3 or 4. The CPE of infected cells was obvious by their appearance and in contrast, there was no pathological change in the mock infected cells (Figure 1C).

The PPV virion with an ovoid shape and spiral crisscross pattern was easily identified by morphological features using EM (Figure 1D). Virus particles in ultrathin sections were observed in the cytoplasm of infected cells at 72 h post inoculation (pi) (Figure 1D).

Immunofluorescence was used to determine the causative agent responsible for CPE, with the virus recognized by 2E4 mAb during cell infection. The images were taken using a Leica fluorescence microscope (Figure 1E).

The target genes in viral DNA samples were detected successfully by PCR. Although all of the target bands appeared, before that there was an interlude during this period. Initially, 3 pairs of synthetic oligonucleotide primers namely B2L-F/R, F1L-F/R, and VEGF-F/R designed according to the NZ2 strain were used for PCRs. Both B2L and F1L were successful but absence of VEGF band was shown. It is not surprising that application of PCR primers (VEGF-F'/R') designed according to the NZ7 strain allows us to detect the VEGF gene. Together, these three bands were corresponding to our expectation with the full-length genes as 1,137, 1,029, and 447 bp, respectively, in a 1.0% agarose gel electrophoresis (Figure 1F). The PCR products were purified and cloned for direct sequencing, and sequence analysis confirmed this ORFV isolate, which was named OV-HLJ05.

### Genetic Characterization of OV-HLJ05

The three genes *B2L, F1L*, and *VEGF* were sequenced to analyze the genetic characterization of OV-HLJ05. Using the Jotun Hein Method in MegAlign program (DNAStar, Inc. USA), a rough outline of the genetic factors of the virus was confirmed.

**Figure 2:**
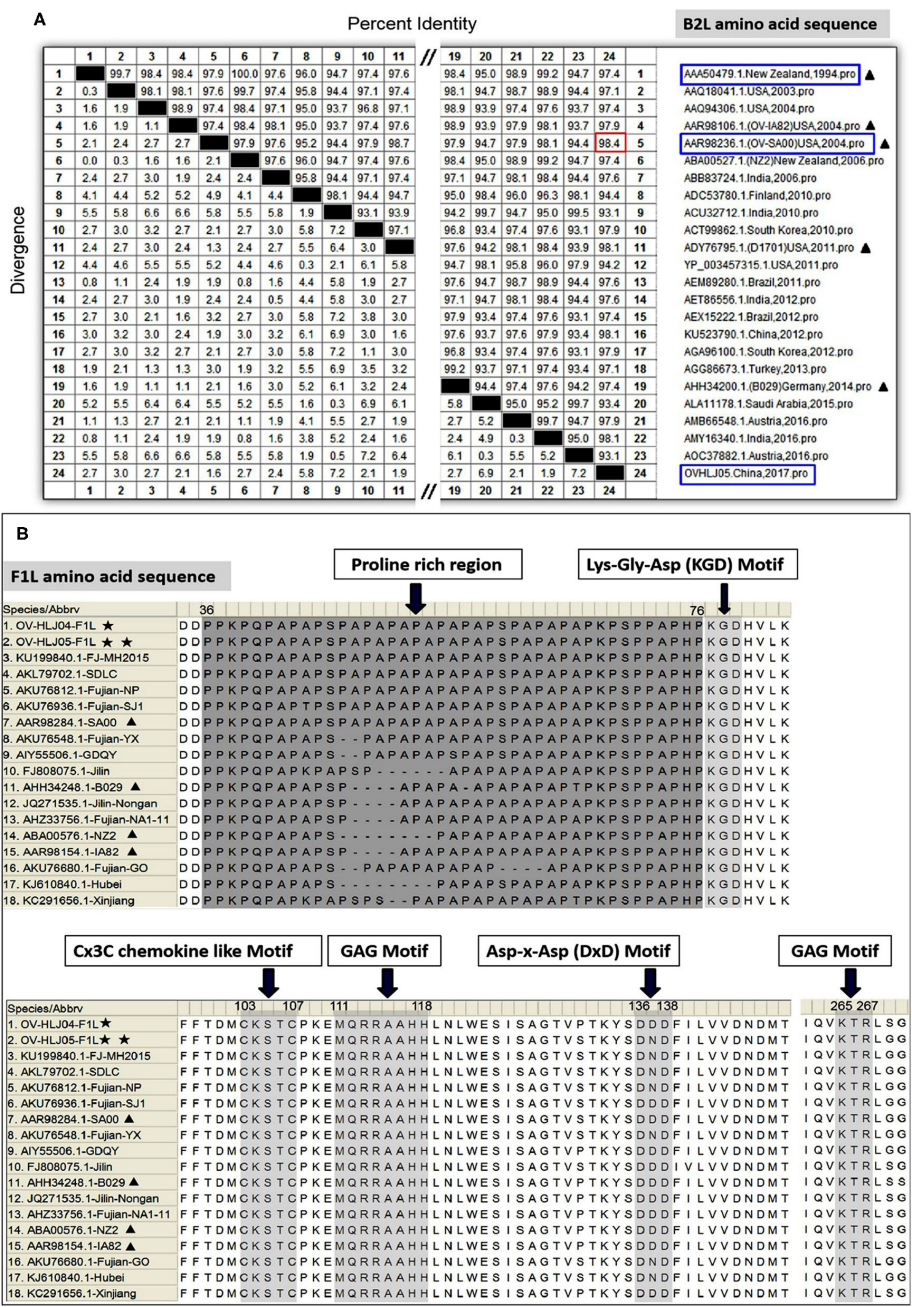
Multiple alignments on the *B2L* genes and on the F1L genes. **(A)** Twenty-four *B2L* gene sequences from the world were used in multiple comparisons and their aa identity was displayed in **(A)**. Black triangles represent five important isolates published previously. Blue boxes represent the isolates which this study investigated. Red boxes represent the maximum among these subjects. **(B)** Fourteen *F1L* gene sequences from China together with NZ2, IA82, SA00, and B029, were used in multiple alignment and the obvious difference was showed at the proline rich regions of N-terminal. Other motifs were essentially conservative (31). One black pentastar indicates the orf virus isolate of previous; Two-black-pentastar indicates the orf virus isolate in this paper.

For the *B2L* gene, a total of twenty-four aa sequences from different sources in the world, including the NZ2 strain (*Accession number*: *AAA50479, ABA00527*); OV-IA82(*AAR98106*); OV-SA00(*AAR98236*); OV-D1701(*ADY76795*); OV-B029(*AHH34200*); OV-HLJ04(*KU523790*); and OV-HLJ05(*MK317955*), were used for alignment in this study. These B2Ls were employed from the GenBank datasets for aa sequence multiple comparison and revealed that all B2Ls were like each other, sharing 93.1 to 98.4% amino acid identity (Figure 2A). It is worth noting that OV-HLJ05 shared 98.4% aa identity with the SA00 strain (*AY386264*) in B2L protein and 97.4% identity with NZ2, so it should be closely related to the SA00 strain genetically.

On comparison of F1L homologs in ORFVs, a total of 18 aa sequences, including NZ2 (*Accession number*: *ABA00576*), IA82 (*AAR98154*), SA00 (*AAR98284*), B029 (*AHH34248*), Chinese OV-HLJ05 (*MK317957*). and OV-HLJ04 (*MK317958*), FJ-MH2015(*KU199840*), SDLC(*AKL79702*), NP (*AKU76812*), SJ1(*AKU76936*), YX (*AKU76548*), GDQY(*AIY55506*), Jilin(*FJ808075*), Nongan (*JQ271535*), NA1-11(*AHZ33756*), GO (*AKU76680*), Hubei (*KJ619840*), and Xinjiang (*KC291656*), were aligned in batches. This study found that the *F1L* gene was highly conserved as well within the ORFV group, except for the proline rich region with a repetitive character in the N-terminal of F1L protein. In addition, several highly conserved motifs mentioned by Scagliarini et al. (37) and Yogisharadhya et al. (31) such as the GAG motif, KGD (Lys-Gly-Asp) motif, KTR motif, D/ExD motif and a Cx3C motif of interest all remained stable in their basic amino acid composition (Figure 2B).

Taken together, the *B2L* gene and the *F1L* gene in all isolates from around the world were relatively conservative in viral genomes.

**Figure 3:**
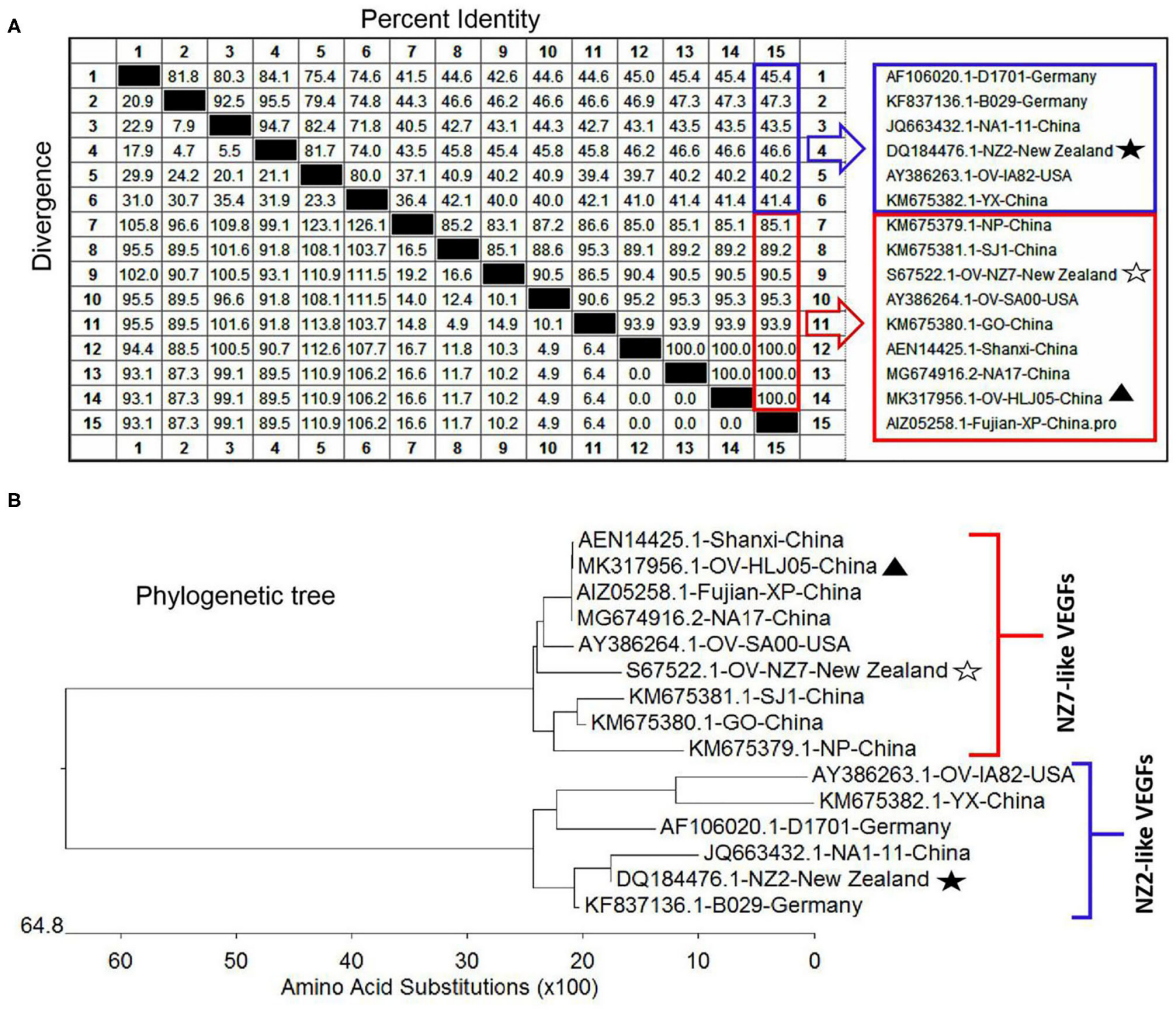
Comparison of different VEGFs with the OV-HLJ05 strain and related sequences published in GenBank. Solid black pentacle denotes the NZ2 strain; hollow black pentacle denotes the NZ7 strain; solid black triangle denotes the OV-HLJ05 strain in this study. **(A)** An alignment of the deduced amino acid sequences of the VEGFs from various sources was generated using MegAlign and Clustal W Method. The aligned sequences are assembled in blue box (low identity to OV-HLJ05) or red box (high identity to OV-HLJ05). **(B)** Predicted evolutionary relationships between the OV-HLJ05 strain and the references in the world mentioned above. They include OV-HLJ05, divided into two groups as the NZ2-like VEGFs (blue half-lattice frame) or the NZ7-like VEGFs (red half-lattice frame).

For the *VEGF* gene, it was also found during the multiple alignment that two clustering groups known as the NZ2- and the NZ7-VEGF like isolates between aa sequences were used in this study. The isolates involved in comparison were ORFV-NZ2 (*DQ184476*), SA00 (*AY386264*), NZ7 (*S67522*), IA82 (*AY386263*), D1701 (*AF106020*), B029 (*KF837136*), NA1/11 (*JQ663432*), NA17(*MG674916*), Shanxi (*AEN14425*), Fujian-XP (*AIZ05258*), GO (*KM675380*), NP (*KM576379*), YX (*KM675382*), and SJ1(*KM675381*). Among them, OV-HLJ05 (*MK317956*) was shown to share 100% identity with the NA17, Shanxi and Fujian-XP strains, which all came from the Jilin, Shanxi and Fujian provinces in China, 94.6% identity with the SA00 strain and 89.2% identity with the NZ7 strain (Figure 3A). The phylogenetic tree showed that the OV-HLJ05 has a highly homologous relationship to SA00 compared with NZ7 despite coming from the same group (Figure 3B). In contrast, the inconsistent amino acid residues in the NZ7 strain occurred about 16 times, compared with eight times in the SA00 strain (data not shown). According to the current alignments, these two groups formed immediately by the program possessed obvious differences in VEGF sequence length between each other. The NZ7-VEGF like group had approximately 150 more residues, but the NZ2-VEGF like group had about 130 residues. Those additional residues in the NZ7-VEGF like individuals were shown as a TATI(L)QVVVAI(L) motif (IR1) and a SSSSS or SSSS motif (IR2) and they occupied two positions front and back in the protein respectively (**Figure 5A**). Insertion of TATI(L)QVVVAI(L) made the first two cysteine positions move back, but the other eight cysteine residue positions have not been impaired by insertion mutation. Residue substitution mutation on the first cysteine residue position, meant that cysteine was replaced by glycine in some NZ2-VEGF like strains including NZ2, D1701, B029, and IA82 (**Figure 5A**), but no mutation on this position was observed in the NZ7-VEGF like individuals. In the NZ7-VEGF like individuals like OV-HLJ05, the serine level has been raised because of the additional SSSSS or SSSS motif (**Figure 5**).

**Figure 4:**
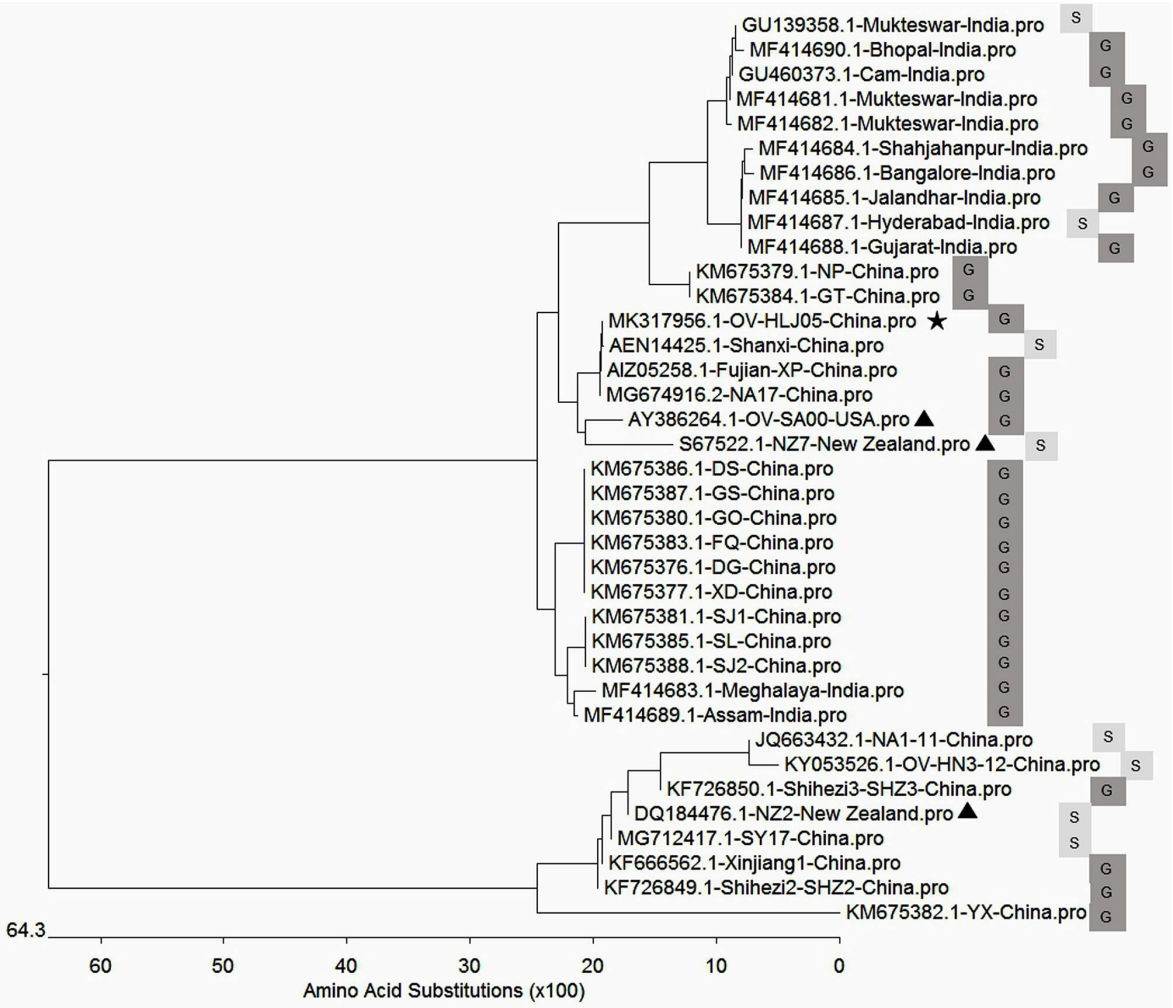
Phylogenetic relationship of VEGFs encoded by Orf virus between the reference strains worldwide and the isolates from China and India. The black triangle indicates reference strains; the black pentacle symbol indicates the OV-HLJ05 strain in this study. Letter S indicates sheep host, and the letter G indicates goat host.

In aa composition, the 37 VEGFs published in GenBank including NZ2, NZ7, SA00, and 22 isolates from China and 12 isolates from India showed two separate parts in the phylogenetic tree map (Table 2). The percentage of the NZ7-like VEGFs have about 79.4% of all sequences derived from China and India, while only 20.6% of sequences have the NZ2-like VEGFs (Figure 4).

### Structural Modeling Implied Heterogeneity Between the Current VEGFs

**Figure 5:**
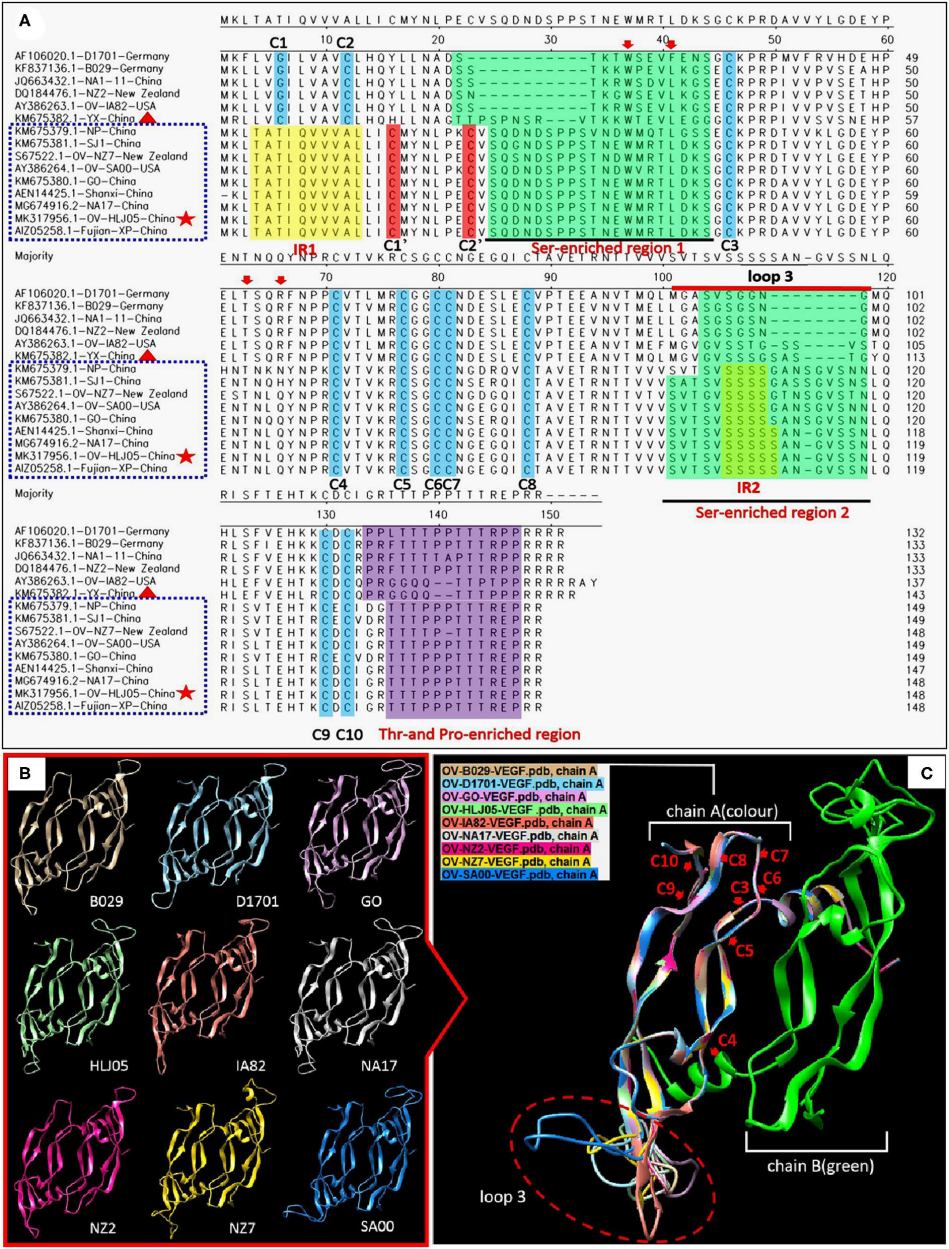
Comparison of amino acid levels between primary and secondary structures of VEGFs. **(A)** Alignment in primary structure of VEGFs. VEGF references of the whole length genome published from GenBank were employed to reflect the structural homology with OV-HLJ05. Important residues matching the consensus of the alignment of the viral VEGFs are shaded in various colors. The two green regions indicate Ser-enriched region I and II; the two yellow regions indicate “insertion mutation” [IRI:TATI(L)QVVVAI(L) motif and IRII:SSSSS or SSSS motif]; the Thr- and Pro-enriched region is shaded in purple (potential *O*-linked glycosylation sites) (33); two cystine knot motifs C1' and C2' are shaded in red whereas the other constitutionally stable ones (C1 to C10) are shaded in blue color. These include the eight cysteines of the cystine-knot motif (15, 33). Loop3 is indicated by a red line. The red arrows indicate binding site of VEGFR-2 (33); dotted blue frames represent the NZ7-VEGF like members, red pentacles represent OV-HLJ05 and red triangles represent YX from China. **(B)** Ribbon representations of the predicted structures of dimer of selected members of the VEGF family are shown, respectively. **(C)** Superimposed structures by members from **(B)**. On the **(A)** chain, cystine residues (C3 to C10) are labeled by red letters, and the loop3-regions in **(A)** are shown by an oval dotted red frame.

**Figure 6:**
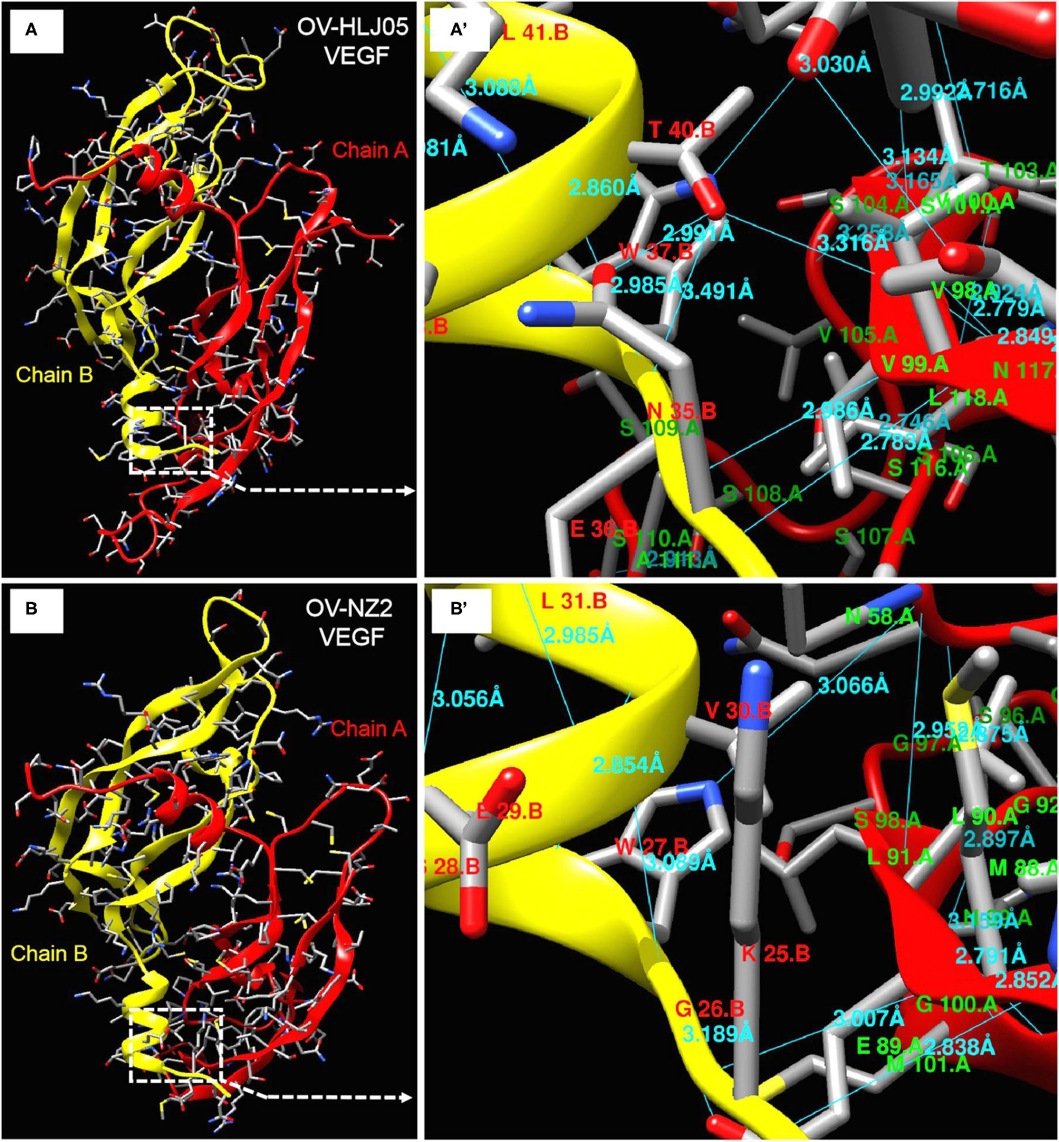
Deeper insight on dimer structures of OV-NZ2 VEGF and OV-HLJ05 VEGF. **(A)** The dimer structure of OV-HLJ05 VEGF is composed of chain A (red) and chain B (yellow). **(A')** showed the enlarged outline of the contact sites of chain A and chain B with more hydrogen bonds. **(B)** The dimer structure of NZ2 VEGF is composed of chain A (also in red) and chain B (also in yellow). **(B')** showed the enlarged outline of the contact sites of chain A and chain B with several hydrogen bonds. The contact parts from **(A',B')** were intuitively compared for finding the inner differences of OV-HLJ05 VEGF and NZ2 VEGF, resulting in distinction on amino acid residue configuration with various hydrogen bond distance in the contact regions.

The predicted structures of the VEGF-variants of ORFVs were determined by comparison to the solved crystal structure of subunits A and B of 2gnn.1 (orf Virus NZ2 Variant of VEGF-E in SWISS-MODEL). Homologous modeling showed maximum heterogeneity at loop three (Figure 5C) and the contact points of chain A and chain B (Figure 6) between the VEGFs. There was more heterogeneity between the NZ2-like VEGFs and the NZ7-like VEGFs, but the essential structure was conservative (Figure 5). In addition, the residues involved in dimerization of chain A and chain B of VEGF protein monomer were from ST^34^NE**W**^**37**^MRT**L**^**41**^DK^43^S^44^G^45^ of chain B in the OV-HLJ05 strain, compared with NT^24^KG**W**^**27**^SEV**L**^**31**^K^32^G^33^S^34^ in the NZ2 strain. Among these two motifs, the “Txx**W**xxx**L**(x)KSG (GS)” motif was a relatively conservative pattern that was probably responsible for dimerization of chain A and chain B and the motifs **W**xxx**L**. Another two motifs, **T**xx**R** in NZ2 and **T**xx**Q** in NZ7, were responsible for binding VEGFR-2 according to Mercer's report, with x representing any amino acid residue. For the dimerization, H-bonds between chain A and chain B, particularly at the binding site, were labeled in different lengths ranging from 2.7 to 3.4 Å (Figure 6).

## Discussion

Orf epidemics are common in the world particularly in developing countries such as China, India, and South Africa but this disease has been largely ignored, due to relatively low mortality rates, or spontaneous recovery (38). In this study, a small outbreak of orf involving a suburban livestock farm in northeast China was investigated, where the Boer goat kid presented for evaluation died. The ORFV was suspected to be responsible for this outbreak and it was confirmed and identified in our laboratory.

As a primary subject of study, the B2L protein of ORFV is a F13L homolog of Vaccinia virus (VACV). The F13L is purportedly required for the efficient formation of enveloped VACV virions (39) and contains the variant HKD (His-Lys-Asp) motif of phospholipases and phospholipid synthases (40, 41), leading to a report of associated lipase activity (42). The B2L protein owns the same HKD variant as in F13L which has an NKD pattern, where the His in HKD is substituted by the Asn both in F13L and B2L, while the detailed biochemical lipase function of B2L protein remains unclear. The *B2L* gene is used for phylogenetic analysis of ORFV (25–28) and PCR by *B2L* has been described previously as an available tool to amplify target DNAs within the PPV genus (21). However, merely to investigate molecular characterization of ORFV isolate, information of the *B2L* gene combined with the *F1L* and *VEGF* genes is necessary for understanding of the virus. Comparative analysis resulted in OV-HLJ05 owning an extensive homologous relationship with the SA00 and the NZ7 rather than the NZ2 in these three genes. Unsurprisingly, OV-HLJ05 has some divergence from other candidates in the *B2L* gene product constitution (see Figure 2A), but this was not enough to affect their conservative nature due to their over 93% identity.

Another evidence of stability is for the F1L protein. Beside the proline rich regions, during the sequence alignment, the study found the functional motifs, which was mentioned by Yogisharadhya's team (31). The similar quality suggested that the F1L was maintaining its multiple roles with intra- and extra-cellular activity during ORFV infection and the largest heterogeneity between these F1L targets was found to be only located in the proline-rich regions. This event was initiated by the natural deletion or loss of individual proline residues in viral generation, but in fact it hardly impairs F1L's functions (37).

Previously, all VEGFs were shown as only 41 to 61% aa sequence identity among PPVs by Delhon et al. (16). In ORFV, genetic consistency presents a polarized distribution, therefore, two typical genotype groups also known as the NZ2- and the NZ7-like VEGFs were presented by Mercer (33). The VEGFs were used as representatives of the diversity analysis within the ORFVs even though this study did not know the scale and distributed situation of the two groups (14, 15, 33). Genetically, it was possible that the NZ7-like VEGF was acquired by ORFV independently of the NZ2 acquisition event and from a different source. The virus with NZ7 VEGF genotype can be found around the world, particularly in India as described by Karki et al. (34) and in China as shown by the results of this study, so the virus may have been selected by adaptation or from host species from distinct environments. Both these VEGF-like ORFVs can stabilize the inheritance of the genome, with the remaining critical issues studied by epidemiology and pathology. The data from this study suggested that OV-HLJ05 strain was closer to the SA00 strain at the aa level particularly in the *VEFG* product (Figures 3A,B). Except for three Chinese isolates including NA17, Shanxi and Fujian-XP, OV-HLJ05 was found to share 95.3% identity of VEGF with the SA00 strain and to exceed 90.5% identity with the NZ7 (Figure 3A). There was increasing evidence that clinical symptoms of the affected kid in this outbreak were similar to reports from Guo et al. (26) with the SA00 strain affecting North American and Texan Boer goat flocks, Hosamani et al. (43), involving the Muk5905 strain in a Mukteswar goat in India, Charles (44) with TZ/BB/13 strain in a Tanzanian goat and Zhang et al. (45) who reported on three strains SDLC, SDTA, and SDJN identified from a Shandong goat in East China, as relevant examples. None of the quoted studies provided any VEGF information.

For investigating the essential divergence between the NZ7-VEGFs and the NZ2-VEGFs, the full length of VEGF primary structures were elaborately arranged in a pool, of course, the region rich in threonine (T) and proline (P) (15) is retained in the C-terminal of all the 33 VEGFs (Figure 5). Despite showing little DNA homology to each other, such as insertion of a TATI(L)QVVVAI(L) motif and a SSSS(S) motif; besides deficiency or substitution, whereas the flanking sequences are over 98% homologous. Depending on the huge homologous nature, the PPVs may be favorably characterized and distinguished with the VEGF-like gene (15). Theoretically, despite the surprising extent of sequence variation among the viral VEGFs, key motifs of structural and functional importance were conserved (33). As both NZ2- and NZ7-like VEGFs have been shown to bind and activate VEGFR-2 functionally, then in the short term their clinical manifestations are nearly indistinguishable (46–49) and structural modeling more objectively reflected heterogeneity between the current VEGFs. The dimerization, contribution of residues for chain B binding to chain A was measured and the Txx**W**xxx**L**(x)KSG (or GS) motif was shared by these two VEGFs. The significant difference found was the number of hydrogen bonds (Figure 6), which implied *in vivo* that their biological activities are not exactly consistent.

An additional discovery during alignment using OV-HLJ05 with the fourteen representative ORFV strains, such as NZ2, NZ7, SA00, IA82, D1701, and B029 and another eight strains from China namely NA1-11, GO, NP, SJ1, YX, NA17, Shanxi and Fujian-XP, showed that the YX strain seemed to be a mid-transition type virus variant between the NZ2 and the NZ7 in VEGF, but currently it still belongs to the NZ2 camp due to the C1 and C2 locations at the N-terminal of protein. This finding is based on observation on the two Ser-enriched regions in VEGF sequences (Figure 5).

Genetic evidence on the *VEGF* genes has already been used to explain the ORFV scenario. An extreme example of application for the *VEGF* gene was to generate a recombinant ORFV known as D1701, a VEGF deletion mutant, by which the influence of ORFV genes in attenuation and virulence were successfully evaluated (50). Available sequence heterogeneity in the *VEGF* gene is likely to be ubiquitous or show individual features of each ORFV isolate from various geographic areas. This variant OV-HLJ05 genetically appears to be consistent to the Shanxi isolate, Jilin-NA17 isolate and Fujian-XP isolate with 100% identity. This condition suggested that the NZ7-VEGF like strain has spread throughout the mainland of China in recent years mainly due to transportation spread and a similar scenario is also presenting in India (34) (Figure 5). Accordingly, most isolates from these two countries have highly homologous VEGF profiles to the NZ7 strain, which seems to favor goats as hosts rather than sheep (Figure 4). This issue needs to be clarified worldwide by extensive epidemiological and statistical investigation.

The animal inoculation experiment showed that GO (NZ7 member) had the strongest virulence, second was YX, but NP and SJ1 showed low virulence (19). The outcome of an experiment sometimes was not consistent with the clinical feature. Previously, ORFV isolated from a goat severely affected by orf had not led to similar severe symptoms in susceptible kids (51). Consideration of the virulence determinants of a virus should not neglect the impacts of the host health status, age, and lifestyle. The impact of endogenous and exogenous factors on susceptibility to ORFV for some goats will probably reflect the host's specific susceptibility toward a certain individual strain, but the fact that NZ7-like viruses mostly come from goats suggests that the species factor should not be neglected in the clinical investigation.

In summary, an elaborated investigation can be used to diagnose the genetic characterization, molecular epidemiology and likely emerging pathogenicity of any new ORFV variant in the field and it is vital that developing countries control such any orf endemic initiated by either of these two genotypes.

## Data Availability Statement

The datasets presented in this study can be found in online repositories. The names of the repository/repositories and accession number(s) can be found in the article/supplementary material.

## Ethics Statement

This article does not contain any studies with human participants or animals performed by any of the authors.

## Author Contributions

YY, ZL, and YC conceived and designed the experiments. XD, YL, JM, and BS performed the experiments. YY, XD, and YC analyzed the data. YY and XD wrote the manuscript and prepared the Figures. YY, XD, and YC checked and finalized the manuscript. YY and ZL provided resources. All authors read and approved the final manuscript.

## Conflict of Interest

The authors declare that the research was conducted in the absence of any commercial or financial relationships that could be construed as a potential conflict of interest.
